# A simple threat-detection strategy in mice

**DOI:** 10.1186/s12915-020-00825-0

**Published:** 2020-07-29

**Authors:** Xing Yang, Qingqing Liu, Jinling Zhong, Ru Song, Lin Zhang, Liping Wang

**Affiliations:** 1 Shenzhen Key Lab of Neuropsychiatric Modulation and Collaborative Innovation Center for Brain Science, Guangdong Provincial Key Laboratory of Brain Connectome and Behavior, CAS Center for Excellence in Brain Science and Intelligence Technology, the Brain Cognition and Brain Disease Institute (BCBDI), Shenzhen Institutes of Advanced Technology, Chinese Academy of Sciences, Shenzhen-Hong Kong Institute of Brain Science-Shenzhen Fundamental Research Institutions, Shenzhen, 518055 China; 2 University of Chinese Academy of Sciences, Beijing, 100049 China

**Keywords:** Automatic behavior detection, Innate fear, Alert range, Freezing, Flight, Rearing

## Abstract

**Background:**

Avoiding danger and accessing environmental resources are two fundamental, yet conflicting, survival instincts across species. To maintain a balance between these instincts, animals must efficiently distinguish approaching threats from low-threat cues. However, little is known about the key visual features that animals use to promptly detect such imminent danger and thus facilitate an immediate and appropriate behavioral response.

**Results:**

We used an automatic behavior detection system in mice to quantify innate defensive behaviors, including freezing, flight, and rearing, to a series of looming visual stimuli with varying expanding speeds and varying initial and final sizes. Looming visual stimuli within a specific “alert range” induced flight behavior in mice. Looming stimuli with an angular size of 10–40° and an expanding speed of 57–320°/s were in this range. Stimuli with relatively low expanding speeds tended to trigger freezing behavior, while those with relatively high expanding speeds tended to trigger rearing behavior. If approaching objects are in this “alert range,” their visual features will trigger a flight response; however, non-threatening objects, based on object size and speed, will not.

**Conclusions:**

These results indicate a simple strategy in mice that is used to detect predators and suggest countermeasures that predators may have taken to overcome these defensive strategies.

## Background

Behaviors that lead to avoidance and escape from imminent danger are fundamental for animal survival [1–3]. However, *flight* behavior costs energy, and hiding in safe places means forgoing opportunities to forage or search for potential mates [4]. As a result, it is of vital evolutionary advantage for animals to detect approaching threats promptly and accurately, while not over-respond to harmless objects.

Visual cues are critical in danger detection. In humans and monkeys, threatening cues in visual stimuli can be detected rapidly without training and may trigger responses even in the absence of any conscious awareness [5]. Visual cues with low spatial frequency are sufficient to trigger affective responses, which are even stronger than responses to high spatial frequency cues [6], suggesting that specific features of visual stimuli, rather than details, are crucial in threat detection. An expanding dark disk called a looming stimulus is widely used in research to simulate an approaching predator or an imminent collision across taxa, including insects [7–9], fishes [10–12], amphibians [6], reptiles [13], rodents [14–16], and humans [17]. Two main features of these looming stimuli are size (visual angle, *θ*) and expanding speed (*dθ*/*dt*). In Drosophila, these two main features are encoded by two distinct neural pathways [8].

The defensive behaviors elicited by looming stimuli are modulated by specific neural circuitry. In insects, looming activates a simple visuomotor neural pathway and generates a fast escape jump [9]. In zebrafish, looming is encoded by a thalamo-tectal pathway and induces a directional startle response to swim away from the source of the stimuli [12]. In rodents, looming is detected by superior colliculus (SC) and triggers freezing and flight behaviors through different neural pathways downstream of the SC [18, 19]. Here, using a series of looming stimuli with different sizes and expanding speeds, we systematically analyzed flight, freezing, and rearing behavior in mice that were induced by these approaching visual cues. We found an “alert range” for specific sizes and expanding speeds, and looming stimulus features within this range elicited a robust flight response.

## Results

### Mice exhibited three typical behaviors in response to looming stimuli

**Fig. 1:**
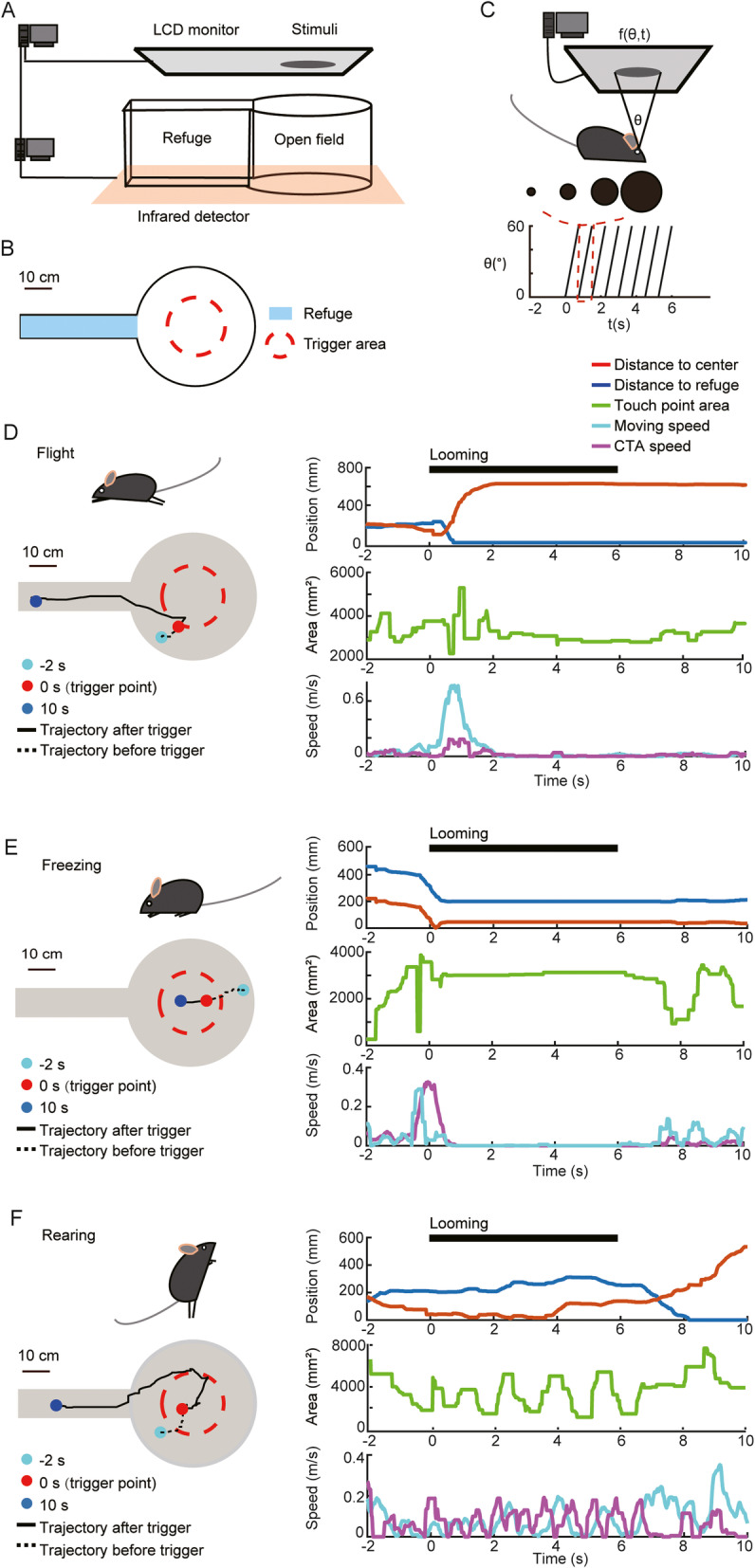
Mice exhibited three remarkable behaviors in response to looming stimuli. **a** Schematic showing the experimental setup: a cylindrical open field, a rectangular refuge, a display monitor above, and an infrared touchscreen frame below. Visual looming stimuli were presented automatically in the open field. **b** Aerial view of the experimental setup showing the trigger area and the refuge. The radius of the trigger area was half the open-field radius. **c** Top: cartoon depicting a mouse presented with a typical looming visual stimulus—a dark disk expanding from 0 to 60°, repeated for 6 s. Bottom: the stimulus can be described by the function *f* (*θ*,*t*) of the time history of the shadow’s size (*θ*). **d** An example of *flight* response from 2 s prior to 10 s after the visual stimulus was triggered. Left: trajectory of a mouse from 2 s before to 10 s after the visual stimulus was triggered. Right, top: distance to the refuge and to the open-field center; middle: touchpoint area; bottom: mouse speed and speed of the change of touchpoint area (CTA). **e** An example of a freezing response. **f** An example of a rearing response

To efficiently screen mouse behavior following visual stimuli, we used an automatic behavior detection system. A mouse was placed in an arena with a circular open field connected adjacently to a narrow alley, which served as a refuge (Fig. 1a, b). A monitor above displayed the visual stimuli. Mouse trajectories were recorded in real time by an infrared touchscreen frame (Fig. 1a). In the center of the open field was a circular trigger zone defined by predefined coordinates within the frame, and thus not visible (Fig. 1b). Looming stimuli were automatically triggered when a mouse walked into this area. The looming stimuli were expanding dark disks with specific size (*θ*) and expanding speed (*dθ*/*dt*), and each visual stimulus was repeated for 6 s without interval (Fig. 1c). Mice were detected by the infrared touchscreen frame in real time as they traversed the arena, and both location and instantaneous cross-sectional area of each mouse were recorded (touchpoint area). Based on the changes in location and touchpoint area, several mouse behaviors were detected, including flight, trotting, rearing, motion in situ, scanning, and freezing (Figs. 1d–f and S1). Among these, flight, freezing, and rearing behaviors have been rigorously studied and could be clearly identified [14, 18–21]. During flight (Fig. 1d and Additional File 1, Figure S1A), individual mice tended to flee to the refuge quickly before the looming stimuli ended. We recorded both distance to the refuge and distance to the center, and in general, the former quickly decreased to zero and the latter increased to the maximum value shortly after mice triggered looming stimuli. The touchpoint area fluctuated during flight and did not change notably when the mouse stayed in the refuge. During freezing (Fig. 1e and Additional File 1, Figure S1B), mice did not show any obvious movement, that is, distance to both the center and the refuge remained unchanged and the touchpoint area was constant. In addition, the speed of the change in touchpoint area over time (CTA speed) and actual moving speed both fell to zero. During rearing (Fig. 1f and Additional File 1, Figure S1C), mice stood on the hind legs and looked upward, and thus, the touchpoint area was reduced for a period of time. In addition, the distance to both the center and the refuge did not change much while the CTA speed increased. In this manner, we analyzed the flight, freezing, and rearing behaviors automatically, based upon the touchscreen frame’s input data.

### The effect of looming speed on looming-elicited behaviors

**Fig. 2:**
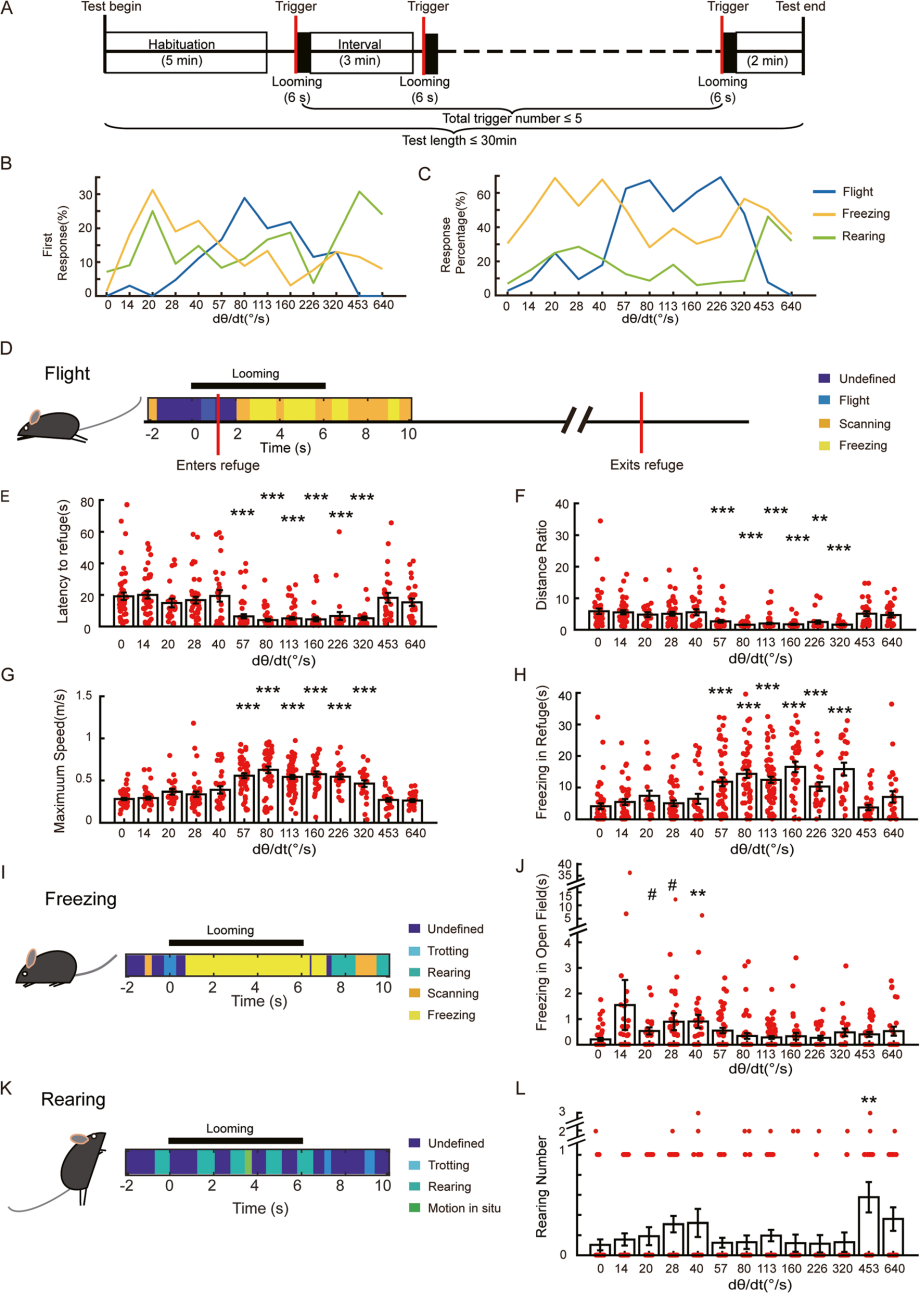
Flight, freezing, and rearing responses induced by an expanding shadow with constant speed. **a** The experiment procedure. After 5 min habituation, a looming visual stimulus lasting 6 s was automatically presented when mice entered the trigger area at walking speed; behavioral responses were identified and recorded automatically. **b** Percentage of flight, freezing, and rearing for each expanding speed for all 1st responses. **c** Percentage of flight, freezing, and rearing for each expanding speed for all responses. **d** Example of automatic behavior identification showing a typical flight response and subsequent freezing in the refuge. **e** Time to reach the refuge after stimulus onset for each expanding speed. **f** Distance ratio for each expanding speed. **g** Maximum speed during stimulus for each expanding speed. **h** Freezing time in the refuge after stimulus onset and before the next entry to the open field for each expanding speed. **i** Example of automatic behavior identification showing typical freezing behavior in the open field. **j** Total freezing time in the open field for each expanding speed. **k** Example of automatic behavior identification showing typical rearing behavior in the trigger area. **l** Total rearing count in the trigger area for each expanding speed. Each dot represents the result of one looming test trial from one animal. The number of mice and trials for each group is summarized in Table 1. Rank sum tests were calculated for comparisons between the experiment and control groups, and the statistical significance between each pair of groups was corrected using the Bonferroni method. Asterisks indicate the level of statistical significance of the fear indices compared to the negative control group (0°/s), ^#^*p* < 0.1, *p < 0.05, **p < 0.01, ***p < 0.001

With this automatic behavior detection system, we investigated the effect of looming stimulus speed on looming-elicited responses in mice. A series of 12 looming stimuli were used. Each looming stimulus expanded with a constant speed [14], from 14 to 640°/s, and expanded from a visual angle of 0 to 60°. We also used a no-looming control group (0°/s). During the first 5 min, each mouse was allowed to explore the entire environment with no visual stimuli. Following this, looming stimuli were triggered when a mouse walked into the trigger zone with a speed below 0.15 m/s. Each mouse was presented with a looming stimulus with one expanding speed, which could be triggered a maximum of 5 times in a 30-min session. The interval between two looming stimuli was no less than 3 min (Fig. 2a). The same behavioral tendencies were evident from the first trial to the fifth (Additional File 2, Figure S2); thus, all trials were treated independently for statistical analyses. The number of mice and trials for each group is summarized in Table 1.

**Table 1:** The number of mice and trials for each group in Fig. 2

	0°/s	14°/s	20°/s	28°/s	40°/s	57°/s	80°/s	113°/s	160°/s	226°/s	320°/s	453°/s	640°/s
**No. of mice**	11	8	6	9	7	14	12	13	10	8	7	6	6
**No. of trials**	48	38	21	42	28	48	46	61	33	26	23	26	25

Based on the ratios of elicited behaviors, looming speed could be divided approximately into three ranges. Looming stimulus speeds in the 57–320°/s range were more likely to trigger flight as a first response than were looming speeds in the 0–40°/s and 453–640°/s ranges and triggered more flight overall during the looming stimulation. Looming speeds in the 453–640°/s range were more likely to trigger rearing than were slower looming stimuli, whereas looming speeds below 57°/s tended to trigger freezing (Fig. 2b, c). Flight responses are shown in Fig. 2d. In the 57–320°/s range, mice reached the refuge significantly faster than the negative control group (Fig. 2e). Trajectories to reach the refuge were evaluated with a distance ratio, which was the ratio of the actual return trajectory to the straight line distance from the mouse (prior to flight onset) to the refuge. In the 57–320°/s range, mice used significantly shorter trajectories to reach the refuge (Fig. 2f) and moved at significantly higher speeds (Fig. 2g) than the negative control group. When hiding in the refuge, mice in the 57–320°/s looming groups exhibited significantly longer freezing times than the negative control group (Fig. 2h), indicating looming-elicited fear. Freezing and rearing behaviors were also investigated in the open field (Fig. 2i, k). For example, following the onset of a looming stimulus, one mouse froze until the looming stimulus ended (Fig. 2i) and another mouse reared four times to observe the looming stimulus (Fig. 2k). The freezing duration was longer for groups in the 14–57°/s range than in the negative control group, while for groups in the 57–320°/s and 453–640°/s ranges, the freezing duration was shorter and was not different to the negative control group (Fig. 2j). Groups above 453°/s had more rearing behavior than the negative control group (Fig. 2l). Thus, flight response was strongly induced by the looming stimuli in the 57–320°/s range, which we refer to as the “flight range.” Freezing responses were more likely to be elicited by looming stimuli no faster than 57°/s, henceforth called the “freezing range.” Finally, rearing responses were largely triggered by looming stimuli above 453°/s, henceforth called the “rearing range.”

### The effect of looming size on looming-elicited behaviors

**Fig. 3:**
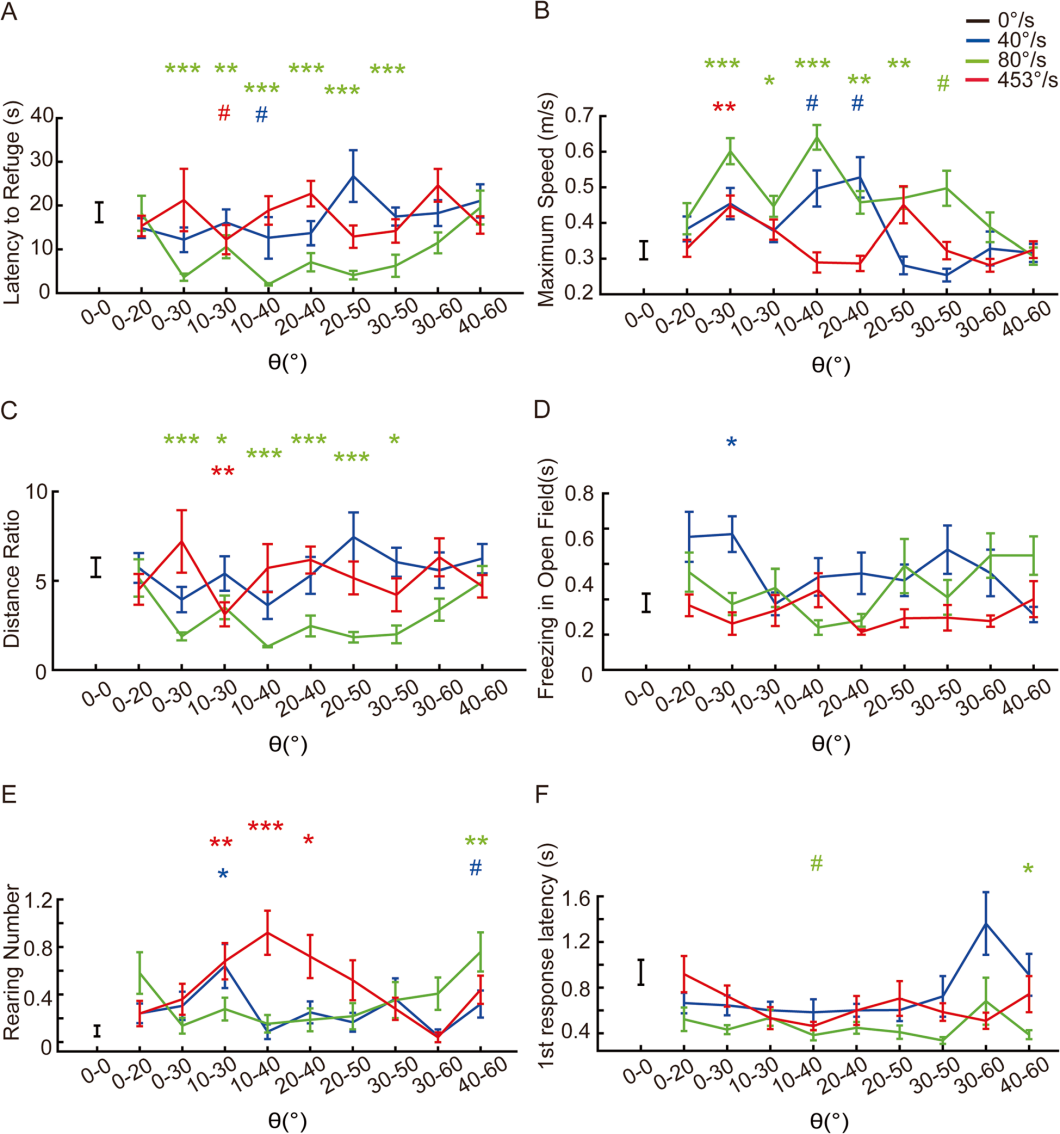
Responses induced by expanding shadows with different changes in expansion size (*θ*). **a** Time to reach the refuge across different size changes. **b** Maximum speed during stimulus across different size changes. **c** Distance ratio across different size changes. **d** Freezing time in the open field across different size changes. **e** Rearing count in the open field across different size changes. **f** Latency to make the first response across different size changes. The number of mice and trials for each group is summarized in Table 3. Rank sum tests were calculated for comparisons between the experiment and control groups, and the statistical significance between each pair of groups was corrected using the Bonferroni method. Asterisks indicate the level of statistical significance of the fear indices compared to the negative control group (0°/s), ^#^*p* < 0.1, *p < 0.05, **p < 0.01, ***p < 0.001

The initial and final sizes of the dark looming disk indicate to the mouse the scale and the distance of the approaching object. We investigated the influence of these size cues to looming-elicited behaviors. We used initial sizes of 0°, 10°, 20°, 30°, and 40°, and final sizes of 20°, 30°, 40°, 50°, and 60°. The change in size (Δ*θ*) was 20° or 30° and a Δ*θ* = 0° group (no looming stimuli) with the initial and final size of 0° was used as a negative control. Looming speeds of 40°/s, 80°/s, and 453°/s were selected from the 3 speed ranges to determine the optimal looming size to trigger defensive behaviors. The 10–40° looming stimulus with the speed in the flight range (80°/s) triggered the strongest flight behavior with the shortest latency to return to the refuge (Fig. 3a), the highest return speed (Fig. 3b), and the shortest trajectory (Fig. 3c). Looming stimuli with speeds of 40°/s or 453°/s did not elicit obvious flight responses irrespective of the disk size (Fig. 3a–c). The 0–30° looming stimulus with the speed (40°/s) in the freezing range triggered significantly longer freezing responses than the negative control group (Fig. 3d). A very long freezing response (36.5 s) was observed in one trial of the 0–60°, 14°/s group, but freezing responses in this group were not significantly different from that in the control group (Fig. 2j). In fact, this speed triggered no significantly longer freezing responses than the negative control group irrespective of the disk size (Additional File 3, Figure S3D). The 0–30°, 10–40°, and 20–40° looming stimuli with the speed in the rearing range (453°/s) triggered significantly more rearing responses than the negative control group (Fig. 3e). Looming with large initial and final sizes, such as 40–60°, mimics a large object close to the mouse; however, these large looming stimuli failed to induce significant flight behavior. In these groups, latency to refuge, return speed, and time spent freezing in the refuge were not different to those in the negative control group (Fig. 3a–c). However, these sizes of looming stimuli were more likely to trigger rearing behavior with speeds in the flight range (Fig. 3e), indicating that mice were curious but uncertain about these stimuli. Looming sizes of 0–20° represent a small object at a long distance and, therefore, did not elicit strong flight responses (Fig. 3a–c). Though not significant, the 0–20°, 40°/s looming stimulus tended to trigger more freezing response (Fig. 3d), and the 0–20°, 80°/s looming stimulus also tended to trigger more rearing behaviors (Fig. 3e). Mice responded faster than the control group to looming stimuli with speeds in the flight range, although this was not statistically significant. There was no correlation between the looming size and response latency (Fig. 3f). These results reveal that mice tend to execute flight behavior in response to an approaching object at a particular scale and distance. In nature, a flight response to a predator in the distance that is not lunging may only draw the predator’s attention. Similarly, if a predator is too close, flight may not be a suitable option because there would be little chance of escape. Taking both flight and rearing into account, the most sensitive looming stimulus size was 10–40°.

### Visual features and mouse behaviors

**Fig. 4:**
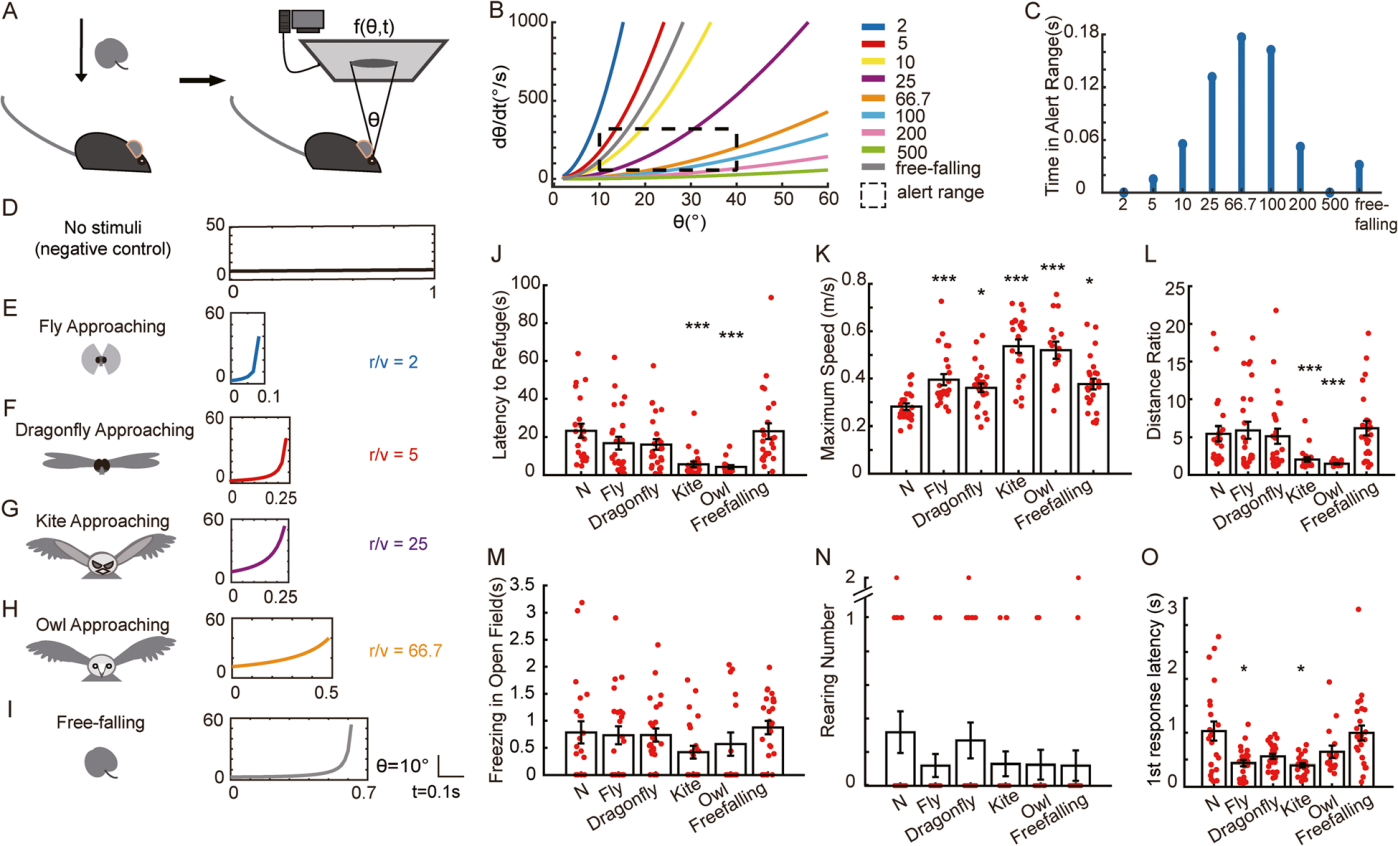
Responses induced by simulative natural visual stimuli. **a** Simulation of an approaching natural object using an expanding shadow. The time history of the shadow’s size (*θ*) is described by the function *f* (*θ*,*t*). **b** The size-expanding speed curves of approaching objects with different radius to velocity ratios (*r*/*v*) and a free-falling apple. The alert range (expanding speed was 57**–**320°/s and angular size was 10–40°) is marked by a black dotted box. **c** Time of each stimulus in the alert range. **d**–**h** Stimuli simulating several natural objects approaching at a constant speed. The first period of each visual stimulus is shown. **i** Stimulus simulating an apple free-falling from the tree. **j** Time to reach the refuge for each visual stimulus. **k** Maximum speed for each visual stimulus. **l** Distance ratio for each visual stimulus. **m** Freezing time in the open field for each visual stimulus. **n** Rearing count for each visual stimulus. **o** The latency to make the first response for each visual stimulus. Each dot in **j**–**o** represents the result of one looming test trial from one animal. The number of mice and trials for each group is summarized in Table 4. Rank sum tests were calculated for comparisons between the experiment and control groups, and the statistical significance between each pair of groups was corrected using the Bonferroni method. Asterisks indicate the level of statistical significance of the fear indices compare against the negative control group (△*θ* = 0°), *p < 0.05, **p < 0.01, ***p < 0.001, ^#^*p* = 0.064

Thus, we found that the most sensitive visual stimulus that would lead to flight was a shadow expanding from 10 to 40°, with an expanding speed between 57 and 320°/s (Figs. 2 and 3). This optimal range for the induction of flight behavior in mice constitutes an “alert range.” However, under natural conditions, the likely angular size of an approaching object nonlinearly increases rather than expands at a constant angular speed. An object could be approximated by a shadow with the angular size *θ*. Then, the process of an approaching object could be described as a nonlinear function *f* (*θ*, *t*) (Fig. 4a). When objects of various scales approach a mouse with constant velocity, the angular size of the object at the retina and the expanding speed are nonlinear functions of time, depending only on the radius to velocity ratio (*r*/*v*) [22]. Approaching process curves were calculated for approaching objects with *r*/*v* values ranging from 2 to 500 ms, resolved into two dimensions: angular size and expanding speed (Fig. 4b). The sections of these approaching process curves that pass through the “alert range” increase first and then decrease as *r*/*v* increases (Fig. 4b, c). Here, several natural visual stimuli were simplified as round objects approaching with constant speed, including a fly in the air (Fig. 4e, *r*/*v* = 2), a flying dragonfly (Fig. 4f, *r*/*v* = 5), an approaching kite (Fig. 4g, *r*/*v* = 25), and an approaching owl (Fig. 4h, *r*/*v* = 66.7) (experimental procedures). The approaching process curves of predator-like stimuli, such as kites and owls, have larger sections in the “alert range” than that of the low-threatening stimuli, including flies and dragonflies, which are almost outside the range (Fig. 4b, c). Besides living beings, a falling object could also produce a looming visual stimulus, and thus, we simulated an apple free-falling from a tree (Fig. 4i) and tested mice responses to all these *natural* stimuli. These stimuli were presented repeatedly for 6 s after being triggered. Mouse behavior with no looming stimuli was used as a negative control (Fig. 4d). For the kite- and owl-approaching groups, latency to refuge was significantly shorter (Fig. 4j), speed was significantly higher (Fig. 4k), and trajectories were significantly shorter (Fig. 4l) than those of the negative control group. In the fly- or dragonfly-approaching groups, latency to refuge and distance ratio were not different to those of the negative control group (Fig. 4j, l). However, in these two groups, the maximum speed to the refuge was significantly higher than that of the negative control group (Fig. 4k), indicating that mice in these groups observed these stimuli and made responses, albeit not flight to the refuge. Remarkably, for the falling-apple stimulus, which is actually a danger cue, its approaching process curve overlaps the “alert range” more so than the dragonfly approaching (Fig. 4b, c). The latency to refuge and distance traveled for mice in the falling-apple group were not different from those in the negative control group, although the moving speed was significantly increased compared with the negative control group (Fig. 4j–l). There was no difference in freezing time in the open field (Fig. 4m) and the number of rearing behaviors (Fig. 4n) between any groups, indicating that these stimuli were not appropriate to trigger freezing or rearing responses. These results suggest that the probability for a visual stimulus to induce flight behavior in mice was positively correlated with the time that the looming stimulus was in the “alert range.” Thus, the threatening level of each visual stimulus could be estimated by the amount of overlap with the “alert range.”

## Discussion

In the laboratory, it is difficult to perfectly simulate natural environments and events to study animal behaviors. It is more convenient and robust to observe behavior under artificial conditions. However, laboratory results only include a small portion or, arguably, perhaps none of the responses an animal may exhibit in the wild, and thus, laboratory conditions may induce cognition and animal behavior different to that in the wild. A systematic study of animal behavioral responses to a series of well-designed stimuli in several typical environments may partly solve this problem, though it also raises experimental difficulty, in addition to the difficulty in data analysis [23]. In natural environments, animals determine how dangerous an approaching object is very quickly. When an object approaches with a constant speed, the evoked shadow expands in a nonlinear manner determined by the size/speed ratio of the object in the animal’s retina. Therefore, nonlinear-expanding dark disks have been used to study defensive behavior in fruit flies and zebrafish [7–12]. In rodents, the looming stimuli used to stimulate defensive behaviors are typically dark disks expanding smoothly, which are more unnatural. However, these stimuli typically trigger robust defensive behaviors [14–16]. We tested two parameters, size (*θ*) and expanding speed (*dθ*/*dt*), which together can determine an expanding process, using an automatic infrared behavior-monitoring system to determine responses to objects that can potentially approach mice.

In the “flight” groups, at least two of the “latency to refuge,” “distance ratio,” and “maximum speed” parameters were significantly different when compared to the negative control group.

We found an “alert range,” in which looming visual stimuli were more likely to trigger flight responses in mice. Visual stimuli outside of the “alert range,” such as a shadow with smaller size and slower expanding speed, possibly representing far-off predators, tended to trigger freezing behaviors in mice. Meanwhile, shadows with a larger size and a faster expanding speed triggered more rearing behavior, suggesting that mice were uncertain about such stimuli (Table 2).

**Table 2:** Mice responses to different looming stimuli

	0–20°	0–30°	10–30°	10–40°	20–40°	20–50°	30–50°	30–60°	40–60°	0–60°
Ctrl*										
14°/s									Rearing	
20°/s	–	–	–	–	–	–	–	–	–	Freezing^#^
28°/s	–	–	–	–	–	–	–	–	–	Freezing^#^
40°/s		Freezing	Rearing							Freezing
57°/s	–	–	–	–	–	–	–	–	–	Flight
80°/s		Flight	Flight	Flight	Flight	Flight	Flight		Rearing	Flight
113°/s	–	–	–	–	–	–	–	–	–	Flight
160°/s	–	–	–	–	–	–	–	–	–	Flight
226^o^/s	–	–	–	–	–	–	–	–	–	Flight
320°/s	–	–	–	–	–	–	–	–	–	Flight
453°/s			Rearing	Rearing	Rearing					Rearing
640°/s	–	–	–	–	–	–	–	–	–	

*Negative control group

–Untested

^#^Approached significance (0.05 ≤ *p* < 0.1)

The visual cues in the alert range are likely to represent common visual features of approaching predators. Therefore, mice should be able to escape from a variety of predators by simply responding to visual cues that correspond to the alert range, rather than by developing specific detection mechanisms for different predators. This simple strategy should contribute to rapid danger detection, although we can only speculate whether this strategy is one that is used in natural conditions. Technical advances, such as virtual reality and behavioral analysis using machine learning, may be useful in determining this in future work [23].

Mice may evaluate threat level using the contrast of the expanding shadow, and neurons in the feed-forward excitatory monosynaptic projection from the medial SC to the dorsal periaqueductal gray (dPAG) may code escape decisions via a synaptic threshold mechanism [21]. In the present study, we demonstrate that visual cues *other* than contrast, specifically, the size (*θ*) and the expanding speed *dθ*/*dt*, also represent the level of danger. These cues are detected by the retina [14] and then induce fast responses via sub-cortical pathways that include the SC [19, 21, 24, 25]. The alert range of crucial visual cues may come from the tuning of looming detection neurons [26]. Combined with recordings of neural responses in looming detection circuits [27], our results may help pave the way to decode the mechanism by which the visual innate fear circuit in mice processes these visual threats.

Moreover, based on our results, a more thorough understanding of predator strategies is required. For example, cats and other feline animals typically slowly approach their prey before a quick attack. This resonates with our result whereby the slowly expanding shadow was unlikely to induce escape behaviors in mice (Fig. 1). In addition, owls prefer to land on a low perch to ambush their prey. Therefore, when they attack, they are already close enough to their prey that little chance of escape is left, which is consistent with our result whereby shadows with large initial size (30~40°) did not have a high probability of inducing flight responses (Fig. 2c, d). These results indicate countermeasures developed by predators against the defensive strategies of their prey and the coevolution between predator and prey.

## Conclusion

Avoiding danger and accessing resources are two conflicting survival instincts. Our results indicate a simple strategy in mice to distinguish approaching predators from other visual cues that signal low threat, which can then facilitate the generation of appropriate behavioral responses. This strategy would be conducive to animal survival in complex natural environments.

## Methods

### Animals

Male C57BL6/J mice (Beijing Vital River Laboratory Animal Technology, China) aged 8–10 weeks were group housed (5 mice/cage) on a 12-h light/12-h dark cycle. All experiments were performed during the light cycle.

### Experimental facility

The arena used to monitor mouse behavior was an open-top acrylic cylinder (50 cm diameter), adjacent to an alley (50 cm × 10 cm), with free access between compartments, and was enclosed by a 30-cm high wall (Fig. 1a, b). Visual stimuli (black) were presented on a 42-in. LCD monitor (refresh rate 60 Hz, AOC) displaying a gray background, positioned 46 cm above the arena floor. The arena was built around an infrared touchscreen frame, which was positioned at the height of a mouse body and recorded the coordinates of the locations visited by each mouse. The recording software was programmed in C++ using visual studio 2015.

### Looming test

Each mouse was habituated to the arena for 5 min, after which the software was set to automatically trigger a looming stimulus when the mouse entered a circular trigger area (diameter 250 mm) with a speed < 0.15 m/s. Each mouse could trigger a maximum of 5 stimulus trials in 30 min with an inter-trial interval ≥ 3 min. Each mouse was tested in only one experimental condition and had 1–5 trials in one session. For each experiment, 5–16 mice were used. The number of mice and trials for each group is summarized in Tables 1, 3, and 4.

**Table 3:** The number of mice and trials for each group in Fig. 3

		0–20°	0–30°	10–30°	10–40°	20–40°	20–50°	30–50°	30–60°	40–60°
Ctrl*	**No. of mice**	11								
	**No. of trials**	43								
**40°/s**	**No. of mice**	6	5	5	5	5	5	5	5	5
	**No. of trials**	29	23	25	23	24	24	22	19	25
**80°/s**	**No. of mice**	8	8	8	7	8	6	6	8	8
	**No. of trials**	31	29	36	26	32	23	17	27	29
**453°/s**	**No. of mice**	5	5	5	5	5	5	5	5	5
	**No. of trials**	25	25	25	25	25	25	25	25	25

*Negative control group

**Table 4:** The number of mice and trials for each group in Fig. 4

	N*	Fly	Dragonfly	Kite	Owl	Free-falling
No. of mice	5	6	6	5	5	6
No. of trials	22	25	26	23	16	25

*Negative control group

Visual stimuli were generated using Psychtoolbox-3 with MATLAB. The visual looming stimulus was a dark shadow-like shape expanding either at a constant angular velocity or with the diameter following the theta-*t* curve simulating natural stimuli (Fig. 4). We approximated (i) an approaching fly as a ball with a radius of 2 mm approaching at 1 m/s from 0.089 m away [28], (ii) an approaching dragonfly as a ball with a radius of 5 cm approaching at 10 m/s from 2.471 m away [29], (iii) an approaching kite as a ball with a radius of 25 cm approaching at 10 m/s from 2.83 m away [30], (iv) an approaching owl as a ball with a radius of 25 cm approaching at 3.75 m/s from 2.5 m away [31], and (v) a free-falling apple as a ball with a radius of 5 cm, an initial velocity of 0 m/s, and an acceleration of 10 m/s^2^ from 2 m away. All visual stimuli were repeated for 6 s.

### Behavioral analysis

All behavioral indices, including the trajectory of the mice to the refuge, speed, time to return to the refuge after stimulus onset (latency to refuge), maximum return speed (maximum speed), freezing time in the open field during looming (freezing in open field) or in the refuge after flight (freezing in refuge), the number of times that each mouse reared in the open field during looming (rearing number), and the latency of each behavior after stimulus onset (1st response latency), were calculated using tracking data obtained from the infrared touchscreen frame. Behaviors were also classified based on tracking data. Flight behavior was defined as moving speed > 0.4 m/s. Trotting behavior was defined as moving speed > 0.2 m/s but < 0.4 m/s for at least 300 ms. Rearing behavior was defined as touchpoint area < 1900 mm^2^ with moving speed < 0.06 m/s and speed of the change in touchpoint area over time (CTA speed) > 0.04 m/s. Motion in situ was defined as moving speed < 0.06 m/s and CTA speed > 0.04 m/s for at least 300 ms while the mouse was not rearing. Freezing behavior was defined as both moving speed and CTA speed < 0.01 m/s for at least 300 ms while touchpoint area > 1900 mm^2^. Scanning behavior was defined as moving speed < 0.04 m/s for at least 300 ms given that the current behavior was not one of the behaviors defined above.

### Statistical analysis

Data are presented as the mean ± SEM. All behavior trials were treated independently for statistical analyses. Rank sum tests were calculated for comparisons between the experiment and control groups, and the statistical significance between each pair of groups was corrected using the Bonferroni method. Statistical analyses were performed using MATLAB. Asterisks indicate the level of statistical significance (*p < 0.05; **p < 0.01; ***p < 0.001).

## Supplementary information

**Additional file 1: Figure S1.** The properties of flight, freezing and rearing responses in mice.

**Additional file 2: Figure S2.** Behavioral tendencies from the first to fifth looming stimulus.

**Additional file 3:Figure S3.** Responses induced by the 14°/s expanding shadows with different changes in expansion size (θ).

## Data Availability

The datasets generated and/or analyzed during the current study are available in the figshare repository, 10.6084/m9.figshare.12504695.

## Abbreviations

SC: Superior colliculus
dPAG: Dorsal periaqueductal gray
CTA: Change in touchpoint area
