# Supplementary material for: A simple threat-detection strategy in mice
Source: BMC Biol. 2020 Jul 29;18:93. doi: 10.1186/s12915-020-00825-0 (PMC7388474; doi:10.1186/s12915-020-00825-0)
Supplement: Supplementary file 1 — Additional file 1: Figure S1. The properties of flight, freezing and rearing responses in mice. [file 12915_2020_825_MOESM1_ESM.docx]

Additional File 1. Figure S1. The properties of flight, freezing and rearing responses in mice.


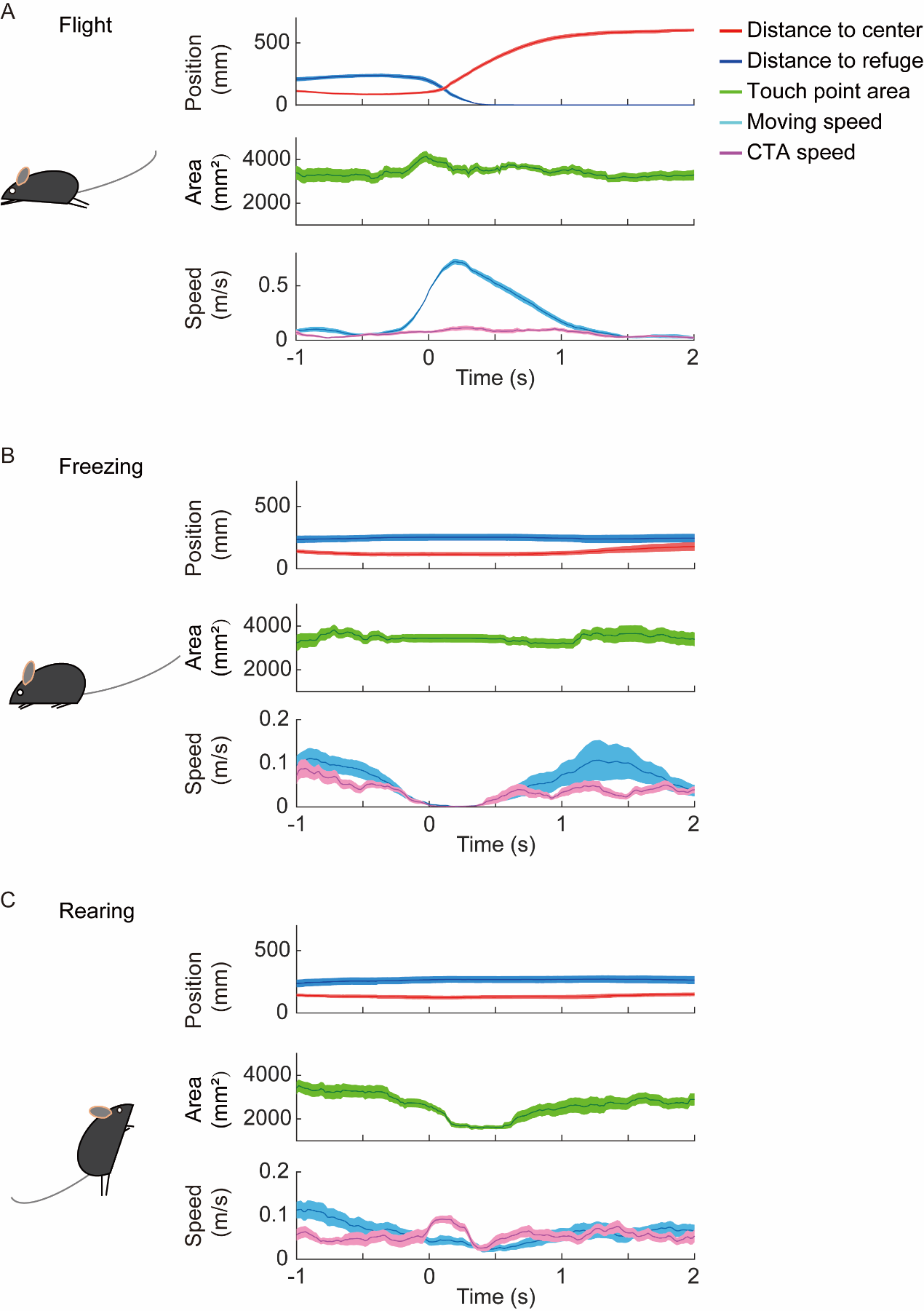


1. Averaged curves from 1 s prior to 2 s after flight response started. *Top*, distance to the refuge and to the open-field center, *middle*, touchpoint area and *bottom*, mouse speed and speed of the change of touchpoint area (CTA). N = 31 flight responses from 12 mice. These mice were presented with 0^°^–60^°^ 80 ^°^/s looming stimuli.
2. Averaged curves in freezing response. N = 19 freezing responses from 7 mice. These mice were presented with 0–60^°^ 40 ^°^/s looming stimuli.
3. Averaged curves in rearing response. N = 19 rearing responses from 6 mice. These mice were presented with 0^°^–60^°^ 453 ^°^/s looming stimuli.

Time zero represents the start time of each response.
