# Supplementary material for: A simple threat-detection strategy in mice
Source: BMC Biol. 2020 Jul 29;18:93. doi: 10.1186/s12915-020-00825-0 (PMC7388474; doi:10.1186/s12915-020-00825-0)
Supplement: Supplementary file 2 — Additional file 2: Figure S2. Behavioral tendencies from the first to fifth looming stimulus. [file 12915_2020_825_MOESM2_ESM.docx]

Additional File 2. Figure S2. Behavioral tendencies from the first to fifth looming stimulus.


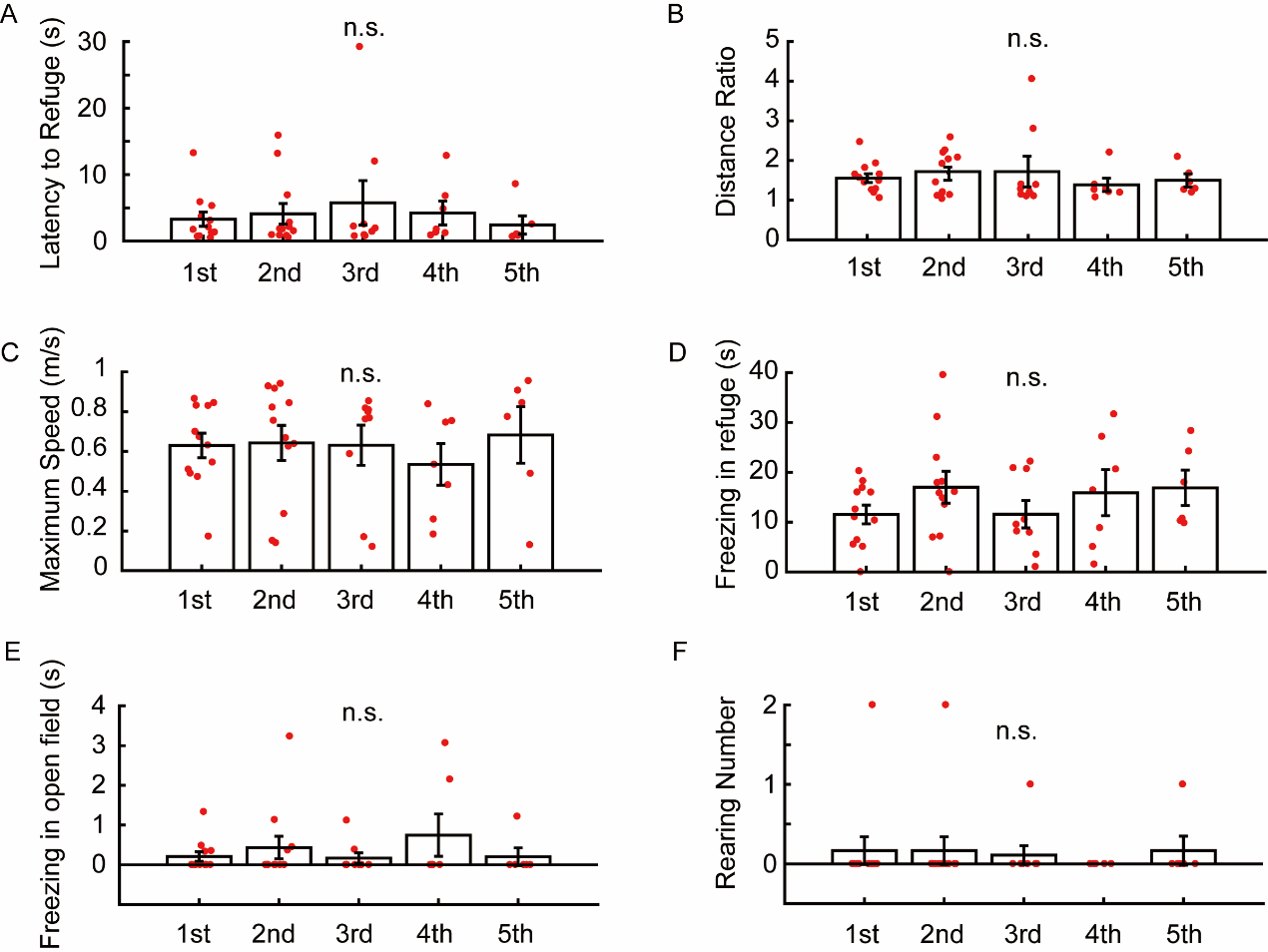


1. Time to the reach the refuge after stimulus onset for each looming stimulus presentation.
2. Distance ratio after each looming stimulus presentation.
3. Maximum speed during each looming stimulus presentation.
4. Freezing time in the refuge after each looming stimulus presentation before the next entry to the open field.
5. Total freezing time in the open field after each looming stimulus presentation.
6. Total rearing count in the trigger area after each looming stimulus presentation.

N =12 mice. The total number of trials from the first looming to the fifth were 12, 12, 9, 7 and 6. Rank sum tests were calculated for comparisons between experiment and control groups and the statistical significance between each pair of groups was corrected using the Bonferroni method. n.s., no statistical significance.
