# Supplementary material for: A simple threat-detection strategy in mice
Source: BMC Biol. 2020 Jul 29;18:93. doi: 10.1186/s12915-020-00825-0 (PMC7388474; doi:10.1186/s12915-020-00825-0)
Supplement: Supplementary file 3 — Additional file 3:Figure S3. Responses induced by the 14°/s expanding shadows with different changes in expansion size (θ). [file 12915_2020_825_MOESM3_ESM.docx]

Additional File 3. Figure S3. Responses induced by the 14 ^°^/s expanding shadows with different changes in expansion size (θ).


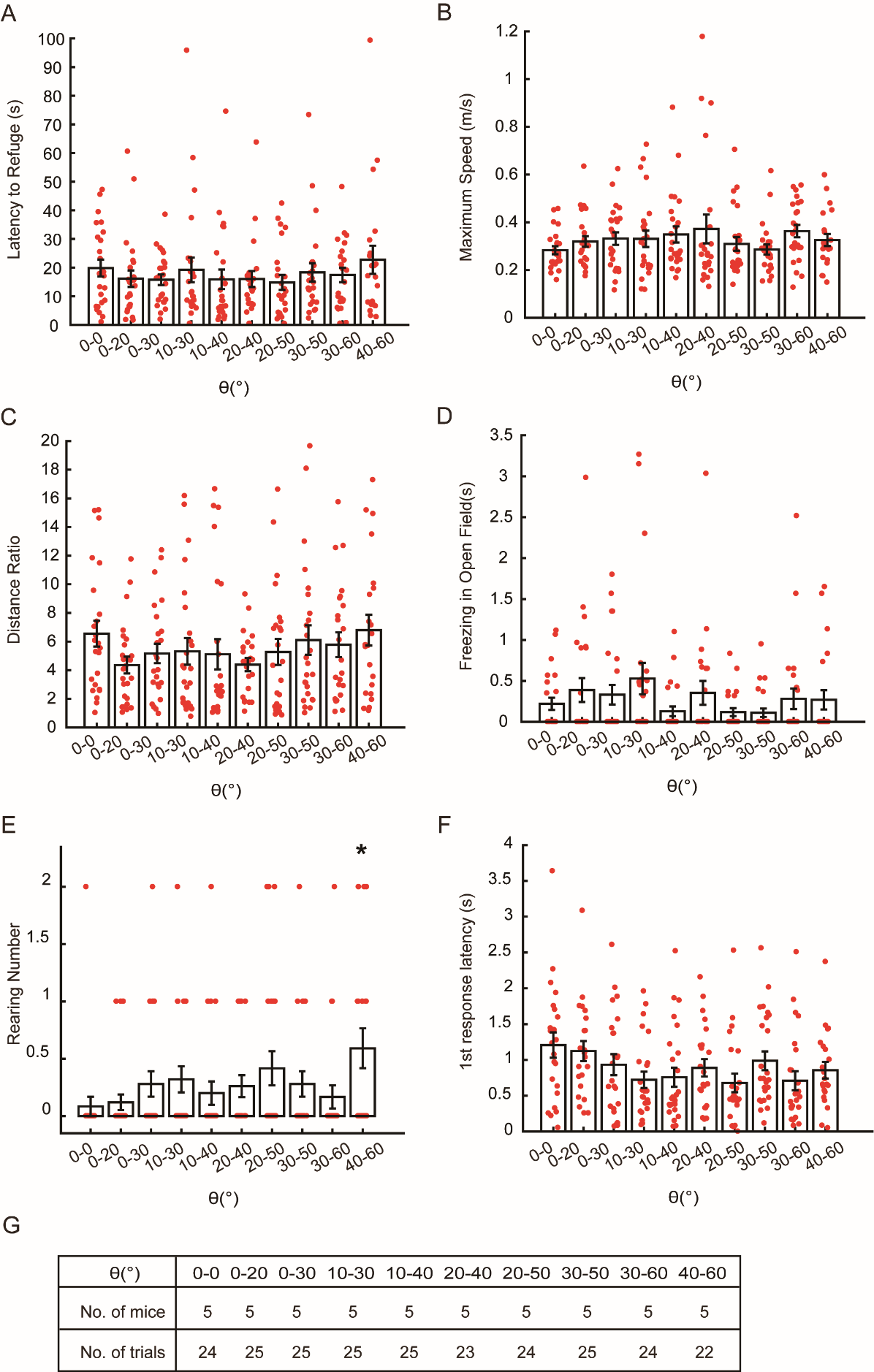


(A)Time to reach the refuge across different size change.

(B)Maximum speed during stimulus across different size change.

(C)Distance ratio across different size change.

(D)Freezing time in the open field across different size change.

(E)Rearing count in the open field across different size change.

(F)Latency to make the first response across different size change.

(G)Number of mice and trials for each group.

Rank sum tests were calculated for comparisons between experiment and control groups and the statistical significance between each pair of groups was corrected using the Bonferroni method. Asterisks indicate the level of statistical significance of the fear indices compared to the negative control group (0 ^°^/s), *p<0.05.
